# The Utrecht cohort for Multiple BREast cancer intervention studies and Long-term evaLuAtion (UMBRELLA): objectives, design, and baseline results

**DOI:** 10.1007/s10549-017-4242-4

**Published:** 2017-04-25

**Authors:** D. A. Young-Afat, C. H. van Gils, H. J. G. D. van den Bongard, H. M. Verkooijen, on behalf of the UMBRELLA Study Group, S. A. Gernaat, M. L. Gregorowitsch, A. M. May, P. H. Peeters, C. C. van der Pol, A. J. Witkamp, R. M. Pijnappel, R. M. Bijlsma, W. Maarse, M. G. Ausems, P. J. van Diest, E. van der Wall, T. van Dalen, I. P. Burgmans, M. A. de Roos, R. C. van Doorn, I. O. Baas, B. van Ooijen, R. Koelemij

**Affiliations:** 1 Department of Clinical Epidemiology, Julius Center for Health Sciences and Primary Care, University Medical Center Utrecht, Utrecht, The Netherlands; 2 Department of Radiation Oncology, University Medical Center Utrecht, Utrecht, The Netherlands; 3 Imaging Division, University Medical Center Utrecht, Utrecht, The Netherlands

**Keywords:** Breast cancer, DCIS, cmRCT, Prospective cohort, Patient-reported outcomes

## Abstract

**Purpose:**

In oncology, RCTs are often beset by slow recruitment, limited generalizability, and strong preferences for interventions by patients and physicians. The cohort multiple randomized controlled trial (cmRCT) is an innovative design with the potential to overcome those challenges. In cmRCT, a prospective cohort serves as an infrastructure for multiple RCTs. We implemented cmRCT in a clinical breast cancer setting by creating UMBRELLA—a large prospective cohort of breast cancer and DCIS patients/survivors.

**Methods:**

For all participants, clinical data and patient-reported outcomes (PROs, i.e., quality of life, fatigue, anxiety and depression, physical activity, work ability, and cosmetic satisfaction) are being collected at regular time-intervals for a period of 10 years. These data are being used both for observational and randomized studies. For each intervention to be tested against standard care, a subcohort of eligible patients is identified within UMBRELLA. From this subcohort, a random sample of patients is offered the intervention. Their outcomes are compared to the outcomes of patients receiving standard care.

**Results:**

So far, between October 2013 and July 2016, we have recruited 1308 participants. In this period, 1308/1486 (88%) patients who were invited for participation in UMBRELLA consented to cohort participation. Of these patients, 1138 (87%) gave broad consent for randomization to future interventions. Return rate for PROs at baseline were 80%, and varied from 67 to 74% during follow-up. Several observational studies—and the first randomized intervention study—are currently ongoing.

**Conclusions:**

Results from UMBRELLA show that this novel study design is feasible and acceptable to patients in a clinical breast cancer setting. We invite researchers who are interested in conducting randomized or observational studies within the UMBRELLA cohort to contact the UMBRELLA scientific advisory board.

## Introduction

With a lifetime risk of one in seven, breast cancer is an important public health concern among women in the Western world [1, 2]. Due to earlier detection and better treatment, breast cancer survival has improved substantially [1–3]. However, current treatment is associated with substantial morbidity, including lymphedema, breast deformities, (chronic) pain, and fatigue. Therefore, new breast cancer treatments should not only focus on further improving (progression-free) survival, but should also aim for good quality of life (QoL), functional outcomes, and satisfying cosmetic results [4].

Interventions aiming to achieve these purposes include minimally invasive treatment of the primary tumor (e.g., axillary irradiation instead of surgery, radiofrequency ablation), as well as lifestyle interventions (e.g., dietary interventions, exercise programs, supportive health apps) [5–7]. Before implementation in routine care, these interventions would ideally be evaluated in randomized controlled trials (RCTs) to confirm whether theoretical benefits translate into actual benefits for patients.

RCTs are the gold standard in comparative research, but often face many challenges. RCTs often are beset by slow recruitment, leading to 40% of cancer trials ending prematurely [8], which is unethical with regards to patients unnecessarily being exposed to potentially harmful or inferior interventions, as well as a waste of time and resources. In the field of breast cancer, the large amount of new interventions entering the market makes it virtually impossible to adequately evaluate each intervention in a separate RCT. It is also complicated to directly compare different interventions tested in separate trials, due to differences in inclusion criteria, outcome measures, and follow-up schemes [9]. RCTs often suffer from limited generalizability due to strict inclusion criteria and selective participation [10]. When highly desired interventions are being evaluated, patients are often disappointed when allocated to the control arm, which may result in drop-out, cross-over, and/or disappointment bias [11]. And lastly, for physicians, the informed consent procedure is cumbersome, as they have to explain (at least) two treatment options that they cannot both with certainty offer to their patients.

In order to deal with these challenges, the cohort multiple randomized controlled trial (cmRCT) design was proposed [12]. In this design, a prospective cohort serves as an infrastructure for multiple RCTs. Advantages of the cmRCT design have been described previously, and include efficient use of control patients, improved comparison between different trialed interventions, enhanced generalizability, and reduced disappointment bias [12, 13].

Clinical and methodological experts in the field of breast cancer combined their knowledge to create a cohort of breast cancer patients according to the cmRCT design—‘Utrecht cohort for Multiple BREast cancer intervention studies and Long-term evaLuAtion’ (UMBRELLA). With UMBRELLA, we aim to

- Generate short- and long-term data on clinical and patient-reported outcomes during and after breast cancer treatment.
- Provide an infrastructure for multiple randomized evaluations of interventions for breast cancer patients and survivors.

In this paper, we describe UMBRELLA’s study design and clinical experiences after 30 months of active recruitment. This paper will serve as the basis for all future observational studies and RCTs using the UMBRELLA cohort.

## Materials and methods

### Enrollment

Patients are recruited at the University Medical Center Utrecht (UMC Utrecht), the Netherlands. All patients with invasive breast cancer and ductal carcinoma in situ (DCIS), who are referred to the department of Radiation Oncology, are eligible for participation in UMBRELLA. Patients with limited understanding of the Dutch language and patients under the age of 18 years are ineligible. Since the UMC Utrecht is the regional center for radiation treatment, UMBRELLA includes patients from secondary and tertiary hospitals. Each year, approximately 575 eligible patients visit the UMC Utrecht for adjuvant radiation treatment of the breast (and axilla).

Before their first visit to the department of radiation oncology, all patients with breast cancer or DCIS receive detailed written information about UMBRELLA. They are scheduled to visit a researcher/research assistant 30 minutes prior to their first appointment with the radiation oncologist. During this research consultation, the researcher/research assistant explains the study in detail, and written informed consent is obtained from those who agree to participate.

The study protocol for UMBRELLA was approved by the Institutional Review and Ethics Board of the University Medical Center Utrecht, the Netherlands.

### Staged-informed consent

UMBRELLA serves as a facility for multiple trials and follows the cmRCT design. In this context, informed consent is obtained through a staged procedure [14]. Before entering the cohort, all patients give written informed consent for collection and use of clinical data. Patient-reported outcomes (PROs) are collected at baseline and at fixed intervals during follow-up.

In addition, patients may give broad consent to be randomly allocated to experimental interventions in the (near) future. Only those randomly allocated to the intervention arm are offered the experimental intervention (which they can accept or refuse). If they accept, additional written informed consent to undergo the experimental intervention will be obtained. Patients who refuse the intervention receive standard care. Patients who are randomly allocated to the control arm also receive standard care, and are not informed about being in the control arm.

Data from all patients may be used for observational studies in UMBRELLA, but only those who provide broad consent for randomization are eligible for participation in RCTs within UMBRELLA. After completion of an RCT within UMBRELLA, all patients—irrespective of participation in the specific study—receive aggregated results.

### Clinical data

Within UMBRELLA, various clinical data are prospectively collected including demographics, tumor characteristics, treatment and toxicity, and imaging data (e.g., mammography, radiotherapy planning computed tomography (CT) scans). Clinical data are captured from electronic medical records, referral letters, and annual reports from the national cancer registry [2].

Sociodemographic data include gender, date of birth, age at diagnosis, highest level of education, postal code (to estimate socioeconomic status), body mass index (BMI) and WHO performance status.

Disease characteristics include method of detection (symptomatic, screening), date of diagnosis, laterality, localization within the breast, classification according to Breast Imaging-Reporting and Data System (BI-RADS) [15, 16], tumor size, nodal status, clinical and pathological stages (classified as American Joint Committee on Cancer c/pTNM classification), multifocality and multicentricity, histologic type, invasiveness, Bloom-Richardson grade, hormone receptor status, and HER-2 status.

Treatment characteristics comprise type of surgery of primary tumor (breast-conserving surgery or mastectomy) and regional lymph nodes (sentinel node biopsy, axillary lymph node dissection, and/or regional radiotherapy), type and timing of reconstructive surgery, surgical margin status (radical, focally irradical, irradical), (neo)adjuvant systemic therapy, radiotherapy parameters (e.g., irradiated volumes, prescribed dose), (surgical) complications, re-admission, and center of surgical treatment.

Toxicity is captured according to the National Cancer Institute’s (NCI) Common Toxicity Criteria for Adverse Events (CTCAE).

Information on recurrence and survival is collected annually by means of (self-reported) questionnaires, the national pathology database (PALGA) and the Central Bureau of Statistics (CBS).

The principal investigators and delegates are responsible for daily cohort management. Data quality is checked periodically. All data are stored and handled according to Dutch privacy law regulations.

### Patient-reported outcomes

We collect PROs by means of validated questionnaires designed to quantify health-related QoL from the patient’s perspective. These questionnaires are sent to patients upon entry into the cohort (baseline), at 3 and 6 months and every 6 months thereafter with a total follow-up of at least 10 years. It takes approximately 20 min to fill out the set of questionnaires at each time point.

Patient-reported information is collected on QoL, fatigue, anxiety and depression, physical activity, work ability, and cosmetic satisfaction through the following questionnaires:

- Quality of Life: European Organization for Research and Treatment of Cancer (EORTC) QLQ-C30, including breast cancer specific module BR23 [17]
- Fatigue: Multidimensional Fatigue Inventory-20 [18]
- Anxiety and Depression: Hospital Anxiety and Depression Scale (HADS) [19]
- Physical activity: QUestionnaire to ASses Health enhancing physical activity (SQUASH) [20]
- Work ability: Work Ability Index (WAI) [21]
- Cosmetic outcome: Cosmetic Evaluation [22].

## Results

So far, between October 2013 and July 2016, we have recruited 1308 participants. In this period, 1308 out of 1486 (88%) patients who were invited for participation in UMBRELLA consented to cohort participation (Table 1). Of those, 1138 (87%) gave broad consent for randomization to future interventions.

**Table 1:** UMBRELLA participation rates and questionnaire return rates between October 2013 and July 2016

	% (*n*/*N*)
Eligible patients	1486
Cohort participation	88% (1308/1486)
*broad consent for randomization*	87% (1138/1308)
Questionnaire return rates^a^	
Baseline	80% (1041/1308)
3 months	74% (868/1178)
6 months	73% (750/1027)
12 months	69% (537/773)
18 months	68% (339/498)
24 months	67% (146/217)

^a^Because this is an ongoing, actively recruiting cohort the denominators decreases

The mean age of cohort participants was 59 years (27–95), 86% were treated with breast-conserving surgery (BCS) and 14% underwent mastectomy. Those who did not provide broad consent for random allocation were slightly older (60 vs. 57 years). Moreover, other differences between patients who provided broad consent for random allocation and those who did not were also marginal (Table 2).

**Table 2:** Characteristics of UMBRELLA participants between October 2013 and July 2016

Characteristics	Full cohort	Consent for future random allocation	No consent for future random allocation	Returned baseline questionnaire	Did not return baseline questionnaire
Number of participants	1047^a^	910	137	838	209
Age at recruitment					
Mean (range)	58 (27–83)	57 (27–83)	60 (28–82)	58 (26–83)	54 (23–82)
Surgery					
Breast conserving	82% (858/1047)	82% (742/910)	83% (114/137)	84% (700/838)	76% (158/209)
Mastectomy	17% (176/1047)	17% (157/910)	14% (19/137)	15% (128/838)	23% (48/209)
ALND only	<0.5% (3/1047)	<0.5% (3/910)	0%	<0.5% (1/838)	1% (2/209)
No surgery	0.5% (6/1047)	<0.5% (4/910)	1.5% (2/137)	0.5% (6/838)	0%
Unknown	<0.5% (4/1047)	<0.5% (4/910)	<0.5% (1/137)	<0.5% (3/838)	0.5% (1/209)
Radiotherapy^b^					
Local	83% (680/815)	83% (584/700)	83% (94/113)	84% (561/666)	80% (119/149)
Loco-regional	14% (116/815)	14% (99/700)	15% (17/113)	13% (88/666)	19% (28/149)
Regional only	1.5% (12/815)	1% (10/700)	2% (2/113)	1.5% (11/666)	0.5% (1/149)
None	<0.5% (3/815)	<0.5% (3/700)	0%	<0.5% (2/666)	0.5% (1/149)
Unknown	0.5% (4/815)	0.5% (4/700)	0%	0.5% (4/666)	0%
Tumor histology^c^					
Ductal	82% (734/899)	81% (634/781)	85% (98/116)	82% (599/731)	80% (135/168)
Lobular	11% (100/899)	11% (89/781)	10% (11/116)	11% (78/731)	13% (22/168)
Ductolobular	3% (27/899)	3% (24/781)	3% (3/116)	3% (25/731)	1% (2/168)
Other	4% (35/899)	4% (31/781)	3% (4/116)	3% (24/731)	5% (8/168)
Unknown	<0.5% (3/899)	<0.5% (3/781)	0%	0.5% (5/731)	0.5% (1/168)
pT-stage					
in situ	12% (109/902)	12% (94/780)	13% (15/120)	13% (93/729)	9% (16/173)
1	58% (542/902)	59% (459/780)	55% (66/120)	59% (427/729)	57% (99/173)
2	20% (180/902)	20% (153/780)	23% (27/120)	20% (142/729)	22% (38/173)
≥3	3% (26/902)	3% (21/780)	4% (5/120)	3% (21/729)	3% (5/173)
X/0	7% (61/902)	7% (53/780)	6% (7/120)	6% (46/729)	9% (15/173)
Screen-detected					
Yes	47% (302/638)	47% (253/543)	48% (45/93)	48% (257/531)	42% (45/107)
No	50% (321/638)	51% (276/543)	51% (47/93)	50% (264/531)	53% (57/107)
Unknown	2% (15/638)	3% (14/543)	1% (1/93)	2% (10/531)	5% (5/107)
EORTC global health status/quality of life (baseline)	74 (18)	74 (18)	75 (17)	74 (17)	75 (19)
EORTC physical functioning (baseline)	85 (16)	85 (16)	85 (15)	84 (16)	84 (15)
EORTC fatigue (baseline)	71 (22)	71 (22)	72 (22)	71 (23)	70 (22)

Percentages may not add up to exactly 100% as a result of rounding

*ALND* axillary lymph node dissection, *pT* pathological tumor size according to TNM classification

^a^These numbers are based on data after linkage with the Dutch Cancer Registry files. Since this process happens annually, not all clinical data for the entire cohort have been obtained. The total amount of collected data may vary per variable as a result of available information at the time of linkage

^b^Loco-regional includes radiation on axillary and/or peri-clavicular lymph nodes

^c^Tumor histology ‘other type’ comprises mucinous, medullary, and metaplastic carcinoma

EORTC scores: Scores range from 0 to 100 and higher scores represent a better health status

Return rates for questionnaires at baseline were 80%, and varied from 67 to 74% during follow-up (Table 1). Sixty percent of patients chose to fill out PROs online, while 40% opted for paper questionnaires.

Descriptive baseline results already provide some insight into patients’ perspectives during and after treatment. Baseline scores for health-related QoL domains are shown in Table 2. Compared to patients who returned the baseline questionnaires, nonresponders were slightly younger (54 vs. 58 years), and a higher proportion of nonresponders were treated with mastectomy (23 vs. 15%) and loco-regional radiotherapy (19 vs. 13%).

Within the cohort, several longitudinal observational studies are investigating PROs in relation to patient, tumor, and treatment characteristics. Studies in progress include, for example, a study on lymphedema of the breast after breast-conserving treatment (incidence, determinants, and the effect of edema on health-related QoL). In another study the association between cardiovascular events and presence of coronary artery calcium on radiotherapy planning CT scans was investigated [23]. This study showed that one in four breast cancer patients planned for radiotherapy have coronary artery calcium, which is known to be a strong risk factor for cardiovascular disease. Within UMBRELLA, Knuttel et al. assessed preferences of breast cancer patients and healthy women regarding new non- and minimally invasive breast cancer treatment options, compared to conventional surgical treatments. These results may be helpful to guide the development of innovative breast cancer therapies and randomized studies to evaluate these novel techniques [24].

Also, the first randomized comparison within UMBRELLA is currently ongoing (FIT trial). The FIT trial evaluates the effect of an exercise program on QoL in breast cancer survivors with low levels of physical activity 12–18 months after diagnosis [25].

## Discussion

### Strengths of UMBRELLA

The major strengths of UMBRELLA are that we systematically invite all eligible patients to participate in UMBRELLA, the high participation rate, the longitudinal capturing of PROs, and the ability to foster multiple trials within a longitudinal cohort. By systematically inviting all eligible patients, and by keeping the physician out of the informed consent procedure, selection is minimized. Due to the high participation rate, UMBRELLA provides a representative study sample.

In UMBRELLA, a wide range of PROs are systematically collected. PROs are becoming increasingly important endpoints to better understand patients’ symptoms, experiences, health-related QoL, and side effects of treatment [4]. Such outcomes will be important when determining which new treatments will be implemented in routine care and will provide valuable input for the process of shared decision-making.

UMBRELLA follows the cmRCT design, which is associated with several advantages. It has the unique ability to facilitate multiple randomized evaluations of (experimental) interventions. Patients may participate in several cmRCTs simultaneously (which may sometimes require stratified randomization if interactions between interventions is to be expected). Direct comparison between interventions is possible, because all trials are conducted within the same study population, making use of the same follow-up scheme and available outcomes [12]. Patients who are not selected for an intervention (the controls) are not informed about interventions under study, which reduces the risk of disappointment bias and contamination compared to classic RCTs. Furthermore, physicians and researchers only explain an intervention that they can *actually* offer to the patient. This reduces the workload of physicians participating in trials, as they only have to explain experimental interventions to patients in the intervention arm.

By adopting a staged-informed consent procedure, we separated consent for cohort participation from consent for accepting interventions after being randomly selected. Instead of receiving a large amount of study information at once, UMBRELLA participants only receive the essential information they need, at the time they need it to make a well-informed decision. This resembles the way information is shared in routine care, thus potentially increasing generalizability to a clinical setting and potentially increasing patients’ ability to process and understand the informed consent procedure.

### Limitations of UMBRELLA

One of the limitations of UMBRELLA is that we only include patients referred for radiotherapy. As a result, around 60% of all invasive breast cancer and DCIS patients are eligible [2]. We have recently obtained ethical approval to expand our cohort to patients without an indication for radiotherapy.

Clinical data collected in this cohort are generated in routine care, and are therefore rather pragmatic. Endpoints for trials within UMBRELLA need to be part of the predefined outcomes being measured for all patients. However, it is possible to collect additional data for specific studies if required.

Since cmRCT is a rather new design, several aspects still need further exploration. For instance, an in-depth evaluation of statistical approaches when running multiple trials with potential for interaction between treatments has not yet been performed.

Finally, the questionnaire return rates slowly decrease over time. This is a problem that many other prospective cohort studies encounter. In our cohort we are actively informing patients about results of studies conducted with cohort data in the hopes of keeping participants actively involved and motivated to return the questionnaires.

## References

[1] National Institutes of Health Consensus Development Conference Statement (1990) Development Program. http://consensus.nih.gov/1990/1990earlystagebreastcancer081html.htm

[2] Netherlands Comprehensive Cancer Organization (IKNL) (2016) https://www.iknl.nl/over-iknl/about-iknl or www.cijfersoverkanker.nl

[3] EBCTCG (2014). Effect of radiotherapy after mastectomy and axillary surgery on 10-year recurrence and 20-year breast cancer mortality: meta-analysis of individual patient data for 8135 women in 22 randomized trials. Lancet.

[4] Fallowfield L, Jenkins V (2015). Psychosocial/survivorship issues in breast cancer: are we doing better?. JNCI J Natl Cancer Inst.

[5] Knuttel FM, Waaijer L, Merckel LG (2016). Histopathology of breast cancer after magnetic resonance-guided high-intensity focused ultrasound and radiofrequency ablation. Histopathology.

[6] Jones LW, Habel LA, Weltzien E (2016). Exercise and risk of cardiovascular events in women with nonmetastatic breast cancer. J Clin Oncol.

[7] Burki TK (2013). Cancer apps. Lancet Oncol.

[8] Young RC (2010). Cancer clinical trials—A chronic but curable crisis. N Engl J Med.

[9] Song F, Altman DG, Glenny AM, Deeks JJ (2003). Validity of indirect comparison for estimating efficacy of competing interventions: empirical evidence from published meta-analyses. BMJ.

[10] Kennedy-Martin T, Curtis S, Faries D, Robinson S, Johnston J (2015). A literature review on the representativeness of randomized controlled trial samples and implications for the external validity of trial results. Trials.

[11] Sedgwick P (2015). Controlled trials: allocation concealment, random allocation, and blinding. BMJ.

[12] Relton C (2010). Rethinking pragmatic randomized controlled trials: introducing the “cohort multiple randomized controlled trial” design. BMJ.

[13] Van der Velden JM, Verkooijen HM, Young-Afat DA, et al (2016) The cohort multiple randomized controlled trial design: a valid and efficient alternative to pragmatic trials? Int J Epidemiol: dyw050 10.1093/ije/dyw050 27118559

[14] Young-Afat DA, Verkooijen HM, van Gils CH (2016). Staged-informed consent in the cohort multiple randomized controlled trial design. Epidemiology.

[15] American College of Radiology (ACR) (2003). Breast imaging reporting and data system atlas (BI-RADS atlas).

[16] D’Orsi CJ, Sickles EA, Mendelson EB (2013). Breast imaging reporting and data system: ACR BI-RADS atlas.

[17] Aaronson NK, Ahmedzai S, Bergman B (1993). The European Organization for Research and Treatment of Cancer QLQ-C30: a quality-of-life instrument for use in international clinical trials in oncology. J Natl Cancer Inst.

[18] Smets EM, Garssen B, Cull A, de Haes JC (1996). Application of the multidimensional fatigue inventory (MFI-20) in cancer patients receiving radiotherapy. Br J Cancer.

[19] Zigmond AS, Snaith RP (1983). The hospital anxiety and depression scale. Acta Psychiatr Scand.

[20] Wendel-Vos GC, Schuit AJ, Saris WH (2003). Reproducibility and relative validity of the short questionnaire to assess health-enhancing physical activity. J Clin Epidemiol.

[21] Ilmarinen J (2007). The work ability index (WAI). Occup Med.

[22] Sneeuw KC, Aaronson NK, Yarnold JR (1992). Cosmetic and functional outcomes of breast conserving treatment for early stage breast cancer. 1. Comparison of patients’ ratings, observers’ ratings, and objective assessments. Radiother Oncol.

[23] Gernaat SA, Išgum I, de Vos BD (2016). Automatic coronary artery calcium scoring on radiotherapy planning CT scans of breast cancer patients: reproducibility and association with traditional cardiovascular risk factors. PLoS ONE.

[24] Knuttel FM, van den Bosch MA, Young-Afat DA (2017). Patient preferences for minimally invasive and conventional locoregional treatment for early-stage breast cancer; a utility assessment. Value Health.

[25] UMBRELLA-FIT trial registration. http://www.trialregister.nl/trialreg/admin/rctview.asp?TC=5482

